# Sustainability of Pain Reduction from Trial Stimulation to Long-Term Follow-Up in Chronic Pain Patients with Spinal Cord Stimulation: A Retrospective Analysis

**DOI:** 10.3390/jcm15218294

**Published:** 2026-10-24

**Authors:** Sibylle Pramhas, Daniela Boryshchuk, Zoltan Micskei, Radoslaw Monte, Sebastian Terner, Andrea Michalek-Sauberer, Sabine Sator

**Affiliations:** 1 Clinical Division of Pain Medicine, Department of Anaesthesia, Intensive Care Medicine and Pain Medicine, Medical University of Vienna, Waehringer Guertel 18-20, 1090 Vienna, Austria; zoltan.micskei@meduniwien.ac.at (Z.M.); radoslaw.monte@meduniwien.ac.at (R.M.); sebastian.terner@gmail.com (S.T.); andrea.michalek-sauberer@meduniwien.ac.at (A.M.-S.); sabine.sator@meduniwien.ac.at (S.S.); 2 Center for Medical Data Science, Medical University of Vienna, Spitalgasse 23, 1090 Vienna, Austria; daniela.boryshchuk@meduniwien.ac.at

**Keywords:** chronic pain, neuropathic pain, spinal cord stimulation (SCS), trial phase, pain level, long-term follow-up, explantation

## Abstract

**Background/Objectives**: Before permanent implantation of a Spinal Cord Stimulation (SCS) system for treatment of chronic pain conditions a trial phase is standard, though its utility is increasingly disputed. Comparisons of long-term to trial-phase pain levels are sparse, and whether trial pain scores predict system explantation risk is unclear. We compare long-term and trial-phase pain levels in SCS treatment and investigate association between trial numeric rating scale (NRS) pain score and explantation. **Methods**: This retrospective, single-centre study analysed patients who underwent SCS implantation with long-term follow-up. Patients were included if baseline and trial NRS diaries were available and trial phase was successful. Missing follow-up NRS values were extracted from patient files, multiple imputation addressing remaining gaps. ROC analysis, log-rank test, logistic regression, and Cox proportional hazards models evaluated association between trial-phase NRS and explantation. **Results**: 71 patients (49.3% female, mean age 53.1 ± 13.0 SD years) were included. NRS reduction during trial was −3.52 from baseline [95% CI: −2.94, −4.09] (*p* < 0.0001). NRS reduction from baseline to long-term follow-up was −1.94 [95% CI: −1.1, −2.74] (*p* < 0.0001). NRS increased by 1.58 [95% CI: 0.78, 2.38] from trial to follow-up (mean follow-up at 4.42 years [95% CI: 3.53, 5.30]) (*p* < 0.0002). Higher trial NRS was associated with higher odds of explantation. (Odds Ratio: 1.38 per one-point NRS increase (95% CI: 1.01–2.04, *p* = 0.0458). **Conclusions**: In SCS there is significant attrition of pain reduction from trial to long-term follow-up. Higher trial NRS showed a possible association with higher odds of explantation. Therefore, pain reduction during trial phase does not necessarily reflect long-term results and utility of trial-phase pain levels for estimation of explantation risk warrants further investigation.

## 1. Introduction

Spinal Cord Stimulation (SCS) is a minimal invasive treatment option for certain forms of chronic neuropathic pain. Common indications include failed back surgery syndrome (FBSS), chronic low back pain with sciatic component and complex regional pain syndrome (CRPS) [1].

However, to date available information on long-term outcome of this treatment modality remains limited.

Accordingly, a recent meta-analysis on SCS in low back-pain concluded that there is little to no information on long-term efficacy [2].

In a randomized controlled trial of SCS in CRPS patients, which compared SCS and concomitant physiotherapy to a control group receiving only physiotherapy, the initially significantly larger pain reduction in the SCS-group which was still present at the two-year follow-up was lost at the 3 and 5 year follow-up [3,4,5].

Therefore, evaluation of stability of pain level reduction evaluated by Numeric Rating Scale (NRS) in long-term SCS-treatment is of interest.

A number of retrospective studies have investigated long-term outcome of SCS [6,7,8,9,10]. Average NRS reductions from baseline ranged between 2.3 and 5 points in follow-up periods between 1 and 10.6 years. Pain levels during trial phase were not reported in these studies and explanted patients were excluded from analysis.

A single retrospective analysis reported on VAS-scores during SCS-treatment in chronic pain comparing the score at 1 month after implantation with score at long-term follow-up. Here a loss of efficacy (1.95 VAS points) was observed at 2 year follow-up [11].

Loss of efficacy constitutes the most frequent reason for SCS system explantation. Therefore, explantation rates, which are reported at up to 38% at 10-year follow-up [12,13] also reflect a decline in efficacy.

Consensus guidelines recommend a stimulation trial phase for all pain indications [14,15] although its utility is not undisputed [16]. While publications on long-term efficacy of SCS evaluated long-term pain levels compared to baseline pain levels, none reported comparisons between pain levels during trial stimulation and long-term pain levels.

If the decision for implantation of an implantable pulse generator (IPG) is based on pain reduction during trial phase, long-term sustainability of this reduction is relevant. Equally, the relationship between pain levels during trial stimulation and explantation rates is of interest.

Therefore, the aim of the current study was to evaluate long-term pain levels in patients with SCS therapy for chronic pain conditions compared to pain levels during SCS stimulation trial phase and the association between pain levels during trial stimulation and probability of explantation.

## 2. Materials and Methods

### 2.1. Methods

This retrospective single-centre study was conducted at the Outpatient Clinic of the Department of Pain Medicine at the Medical University of Vienna after approval by the local ethics committee (Ethics Committee of the Medical University Vienna, Borschkegasse 8b/E06, 1090 Vienna, Austria; Date of registration: 22 September 2023; Registration Number: 1292; Investigator: Sibylle Pramhas, M.D.). The study is reported in accordance with the Strengthening the Reporting of Observational Studies in Epidemiology (STROBE) guidelines for cohort studies. The completed STROBE checklist is provided as Supplementary Material.

As part of our quality control procedure, data from patients, who underwent a SCS trial stimulation at our centre between 2001 and 2021 was reviewed in the electronic patient data base of our centre. Patients were contacted by telephone and asked to complete a follow-up pain diary over a period of one week.

Patients with chronic pain indications (chronic low back pain with leg pain, complex regional pain syndrome, failed back surgery syndrome, polyneuropathy, other neuropathic pain conditions) were eligible for spinal cord stimulation. All patients underwent extensive evaluation prior to SCS-implantation including neurological and psychological assessment. Psychological assessment was targeted to exclude factors that constitute contraindications for invasive therapy such as severe psychiatric illness and current litigation.

SCS-systems manufactured by Medtronic (Minneapolis, MN, USA), Boston Scientific (Marlborough, MA, USA), Abbott (Chicago, IL, USA), Advanced Neuromodulation Systems (Plano, TX, USA), St. Jude Medical (Saint Paul, MN, USA) and Nevro (Redwood City, CA, USA) were used.

Trialling and implanting of the SCS-systems at our centre were performed by a total of 4 physicians.

Octopolar SCS-leads were placed under local anaesthesia and analgosedation in the operating theatre. The tips of the leads were initially advanced under fluoroscopic guidance to the level of Th8, if the painful area was located in the lower extremities and to C2/3 if pain was located in the upper extremities. Intraoperative stimulation was then performed and the final placement of the tips of the leads adjusted according to patient-reported coverage of painful area. Patients were required to confirm at least 80% coverage of the painful area by stimulation-paraesthesia intraoperatively. Leads were then anchored at the fascia and connected to an external stimulator device through an extension lead.

When using a SCS-System manufactured by Nevro (Redwood City, CA, USA) the tips of the leads were advanced to Th8 without further adjustment.

The level of lead tip placement for the included patients (*n* = 71) is given in Supplementary Table S1.

Baseline NRS score was documented over a period of 1–10 days using a pain diary (6 NRS scores/day).

A trial stimulation period of 7–15 days was performed in all patients. NRS scores during trial stimulation were documented in a pain diary (6 NRS scores/day). NRS during trial stimulation was defined as the mean of all NRS values documented during the last 3 days of the trial stimulation phase.

After surgery, individual programming of the device to achieve optimal pain reduction was performed. 61 patients were treated with tonic SCS. 10 patients, who received a SCS-System manufactured by Nevro (Redwood City, CA, USA) were treated with high-frequency stimulation.

Trial stimulation was defined as successful when a ≥50% NRS reduction from baseline was achieved and/or reduction in pain medication, improved quality of life and function were reported. Therefore, patients with ≤50% NRS reduction during trial stimulation were still eligible to receive an IPG if the other criteria were fulfilled.

If the trial stimulation was successful an implantable pulse generator (IPG) was implanted subcutaneously typically in the lower abdominal wall or the upper outer quadrant of the buttock and connected to the stimulator leads. If trial stimulation was unsuccessful the leads were removed. Patient data was included in the current analysis if a pain diary (numeric rating scale (NRS) score) was available at baseline and during trial stimulation and if the patients had received an IPG. During follow-up, available NRS measurements were extracted from the patient records and ordered chronologically as intermediate follow-up measurements; their number and timing varied between patients. Long-term NRS was obtained from the follow-up pain diary when available. In patients who underwent explantation or died before long-term follow-up, NRS measurements documented before these events were retained as observed intermediate measurements, while long-term NRS remained missing.

Opioid use and intake of other analgesic medication was also extracted from the patient files at baseline and follow-up.

### 2.2. Statistical Analysis

Continuous variables were summarised as means ± SD or medians (IQR); categorical variables as counts and percentages. Baseline characteristics were tabulated for included and excluded patients, and stratified by explantation status. Group differences were assessed using the Mann-Whitney U test and chi-squared test, with post-hoc pairwise comparisons where applicable. Fischer’s exact test was used when expected cell counts were small.

### 2.3. Primary Analyses

Of 71 patients with baseline and trial-stimulation NRS measurements who had received an IPG, later follow-up showed considerable missingness. The primary analysis used a linear mixed-effects model with NRS as outcome and time point as a categorical fixed effect (baseline as reference; trial stimulation and long-term follow-up as subsequent time points), plus a patient-specific random intercept. Estimated means and model-based differences were reported with 95% CIs and *p*-values. A pre-specified contrast tested whether the change from baseline to long-term follow-up differed from the change to trial stimulation. Two-sided *p* < 0.05 was considered significant.

Missing values within the longitudinal follow-up NRS sequence were addressed using multiple imputation (MI). Baseline and trial-stimulation NRS were available for all included patients and were therefore not imputed. Missing intermediate and long-term follow-up NRS values were imputed under a Missing At Random (MAR) assumption using a two-level normal model, incorporating baseline covariates and lagged NRS values. Plausibility was assessed via density plots, trace diagnostics, and a mask-and-recover experiment (see also Supplementary Material S2.2). Twenty imputed datasets were analysed separately and combined using Rubin’s rules.

### 2.4. Secondary Analyses

Explantation event was examined using logistic regression (univariable and multivariable) and Cox proportional hazards models. Trial-stimulation NRS was the primary predictor; multivariable models were adjusted for sex, age, indication group, stimulation pattern, number of revisions, and baseline NRS to account for potential confounding. Time to explantation was calculated from implantation; patients without explantation were censored at the last available clinical follow-up. Due to sparse events, Firth-penalized methods were applied to both logistic and Cox regression to reduce small-sample bias.

### 2.5. Exploratory Analyses

Kaplan-Meier curves visualised time to explantation by trial-stimulation NRS group. An optimal cut-off of 4.1 was identified via maximally selected rank statistics (see also Supplementary Material S2.1, Figure S1); for interpretability, patients were grouped as NRS 0–4 versus >4–8, with group differences assessed by log-rank test. Time to explantation was additionally visualised separately for included and excluded patients. ROC curve analysis evaluated the ability of percentage NRS reduction from baseline to trial stimulation to discriminate between patients with and without subsequent explantation, with PPV and NPV reported at the selected threshold.

Given the exploratory nature of these analyses, no adjustment for multiplicity was performed; *p*-values are therefore reported descriptively and should be interpreted together with effect estimates and 95% confidence intervals.

### 2.6. Sensitivity Analyses

To address potential Missing Not At Random (MNAR) missingness following explantation, a sensitivity analysis excluded explanted patients prior to imputation. This restricted analysis was compared with the full imputed analysis as a robustness check. Among non-explanted patients, long-term non-response was considered less likely to represent informative missingness. As no clear systematic trajectory differences were observed between patients who returned a NRS follow-up diary and those who did not, non-explantation-related missingness was considered compatible with the MAR assumption conditional on baseline covariates and available NRS history.

To evaluate potential confounding by implantation era, additional sensitivity analyses were performed. Patients were stratified into two subgroups based on the year of SCS implantation: 2001–2011 and 2012–2021. The previously generated 20 multiply imputed datasets were reused, and linear mixed-effects models with patient-specific random intercepts were fitted separately for each subgroup. Pooled estimates of mean NRS at baseline, trial stimulation and long-term follow-up were calculated using Rubin’s rules. Explantation-free survival was estimated using the Kaplan–Meier method and compared between subgroups using the log-rank test.

All analyses were performed in R version 4.5.1.

## 3. Results

### 3.1. Study Population

184 files of patients who underwent trial stimulation were reviewed in the electronic patient data base of our centre. Four patients were incorrectly entered twice in the patient data base, in 9 patients no data were available in the patient data base. Therefore, data were available in 171 trialled patients.

16 patients had a negative trial resulting in lead removal. (NRS diary available during baseline and trial stimulation *n* = 4; No baseline and/or trial stimulation NRS available *n* = 12)

In the 4 patients, who failed trial and had NRS baseline and trial diaries no long-term follow-up was available.

There were 155 (*n* = 171-16) patients in the cohort, who received an IPG. In these patients an NRS diary was available during baseline and trial stimulation in 71 cases and not available in 84 cases. Therefore, a total of 71 patients were included in the final analysis (pain dairy available at baseline and during trial stimulation and received an IPG).

**Figure 1:**
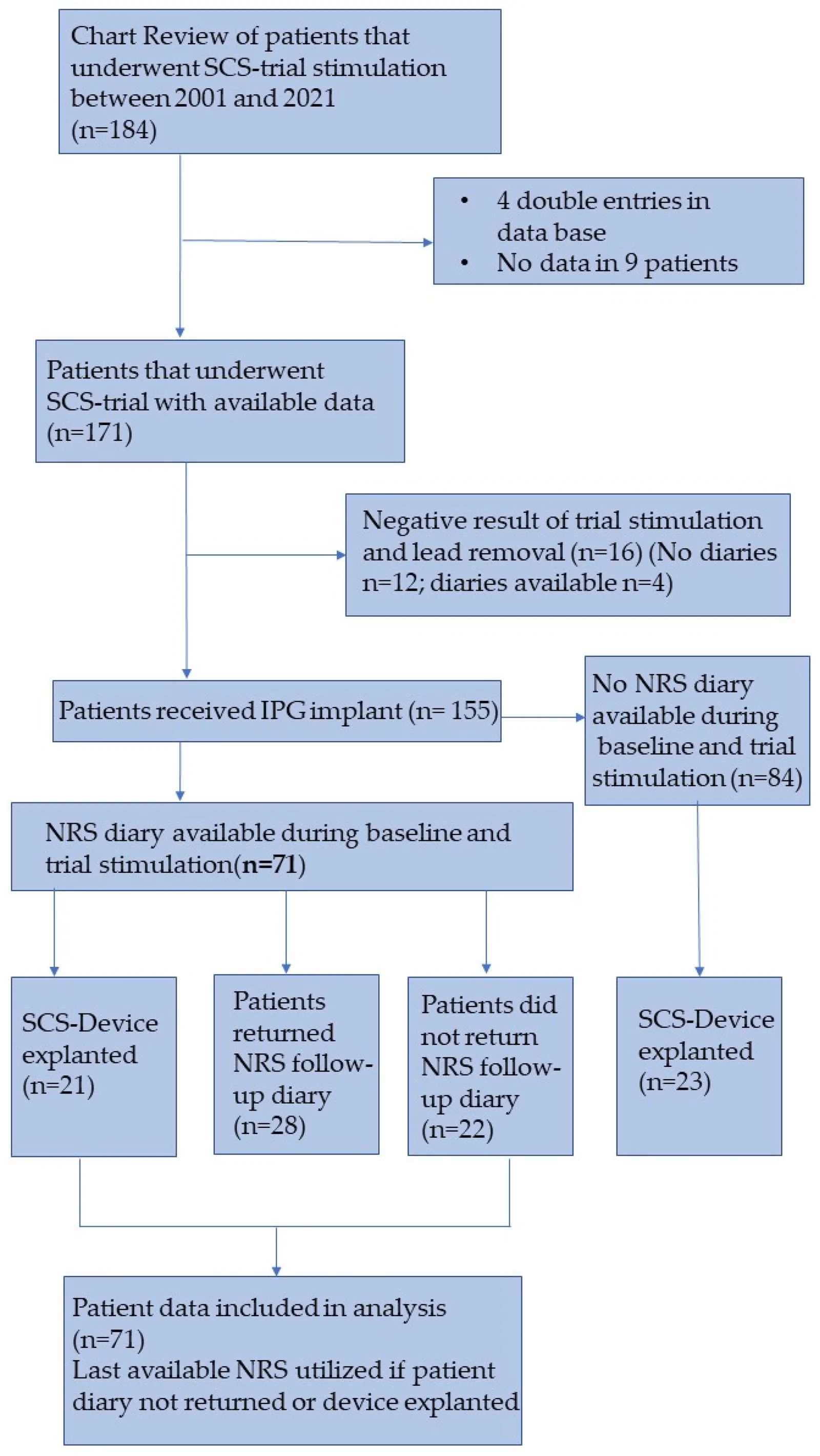
Flow Diagram depicting Inclusion of Patient Data. SCS = Spinal Cord Stimulation; NRS = Numeric Rating Scale; IPG = Implantable Pulse Generator.

Inclusion of patient data is depicted in Figure 1. Patient baseline characteristics for the entire cohort as well as the patients included in the analysis are summarized in Table 1.

Indications for SCS-therapy in included patients, excluded patients and entire cohort are given in Table 1. Failed Back Surgery Syndrome (FBSS) was significantly more frequent in included than excluded patients while ‘other’ indications were significantly more frequent in excluded than included patients.

Within the included sample, NRS at baseline, during trial phase and at long-term follow-up did not differ significantly between FBSS and the remaining indications. There were also no significant differences between ‘other’ indications and the remaining indications. (Supplementary Table S2).

Of 71 eligible patients, 28 (39.4%) returned a NRS follow-up diary. Women were more frequent among patients who did not return a NRS follow-up diary (60.5% vs. 32.1%; *p* = 0.037), whereas indication, age and baseline NRS did not differ significantly (Supplementary Table S3). In patients who returned a NRS follow-up diary, mean NRS did not differ significantly between women and men at baseline, trial and follow-up.

Frequencies of vendor use were as follows: Boston Scientific, *n* = 23 (32.4%); Medtronic, *n* = 21 (29.5%); Nevro, *n* = 10 (14.1%); St. Jude Medical, *n* = 8 (11.3%); Abbott, *n* = 8 (11.3%); Advanced Neuromodulation Systems, *n* = 1 (1.41%).

Overall, the mean time from implantation to last observation (*n* = 71) was 4.42 years [95% CI: 3.53, 5.30].

### 3.2. Explantation Rate

Explantation occurred in 44 of the 155 patients who received an IPG resulting in an explant rate of 28.4% for the entire cohort. The mean time to explantation was 5.96 ± 4.06 years.

In the 71 patients included in the long-term analysis, the SCS-device was explanted in 21 cases (29.6%). This did not differ significantly from the explant rate in the entire cohort (*p* = 0.89). Also, explant rate did not differ significantly between the included patients (21/71; 29.6%) and the excluded patients (23/84; 27.4%) (*p* = 0.9).

The mean time to explantation in the included patients was 4.10 ± 2.40 years.

There was no significant difference in progression to explantation between the included (*n* = 71) and excluded (*n* = 84) patient group (log-rank test, *p* = 0.2). The corresponding Kaplan Meier plot is given in Supplementary Figure S5.

In included patients no significant difference in the cumulative probability of explantation was observed between implantation periods (2001–2011 vs. 2012–2021; log-rank test, *p* = 0.58). Explantation occurred in 9/16 (56.3%) and 12/55 (21.8%) patients, respectively, with median times to explantation of 46.5 and 40.0 months.

Reasons for device explantation in the included patients were: Inadequate pain relief (*n* = 17; 81.0%), other pathology or intervention necessitating removal (*n* = 2; 9.52%), infection/IPG perforation (*n* = 2; 9.52%).

Reprogramming attempts were documented for all patients explanted due to loss of efficacy [median *n* = 4 attempts, Minimum 3; Maximum 5].

### 3.3. Mean NRS Levels at Baseline and During Trial Phase

Mean NRS scores at baseline and during trial phase were 6.84 [95% CI: 6.41, 7.27] and 3.32 [95% CI: 2.90, 3.75] respectively.

Mean percentage of pain reduction from baseline to trial phase was 50.5% [95% CI: 44.0%, 56.9%].

### 3.4. Long-Term NRS Levels

Long-term NRS level was first assessed in patients who returned an NRS diary at follow-up (*n* = 28) In this group mean time from implantation to long-term follow-up was 6.40 years [95% CI: 4.61, 8.20] and mean NRS score at follow-up was 4.17 [95% CI: 3.52, 4.83].

Here, NRS reduction from base line to trial phase was −3.52 [95% CI: −2.94, −4.03] (*p* < 0.0001), while NRS reduction from baseline to long-term follow-up was −2.67 [95% CI: −1.95, −3.38] (<0.0001).

Therefore, NRS increased by 0.850 [95% CI: 0.130, 1.57] points during the mean follow-up period of 6.40 [95% CI: 4.61, 8.20] years. This increase in NRS was statistically significant. (*p* < 0.05).

Subsequently long-term NRS-level was evaluated in all patients for whom an NRS diary at baseline and follow-up was available employing MI (*n* = 71). The mean time to follow-up was 4.42 years [95% CI: 3.53, 5.30]. Mean NRS score at follow-up was 4.90 [95% CI: 4.18, 5.63].

**Figure 2:**
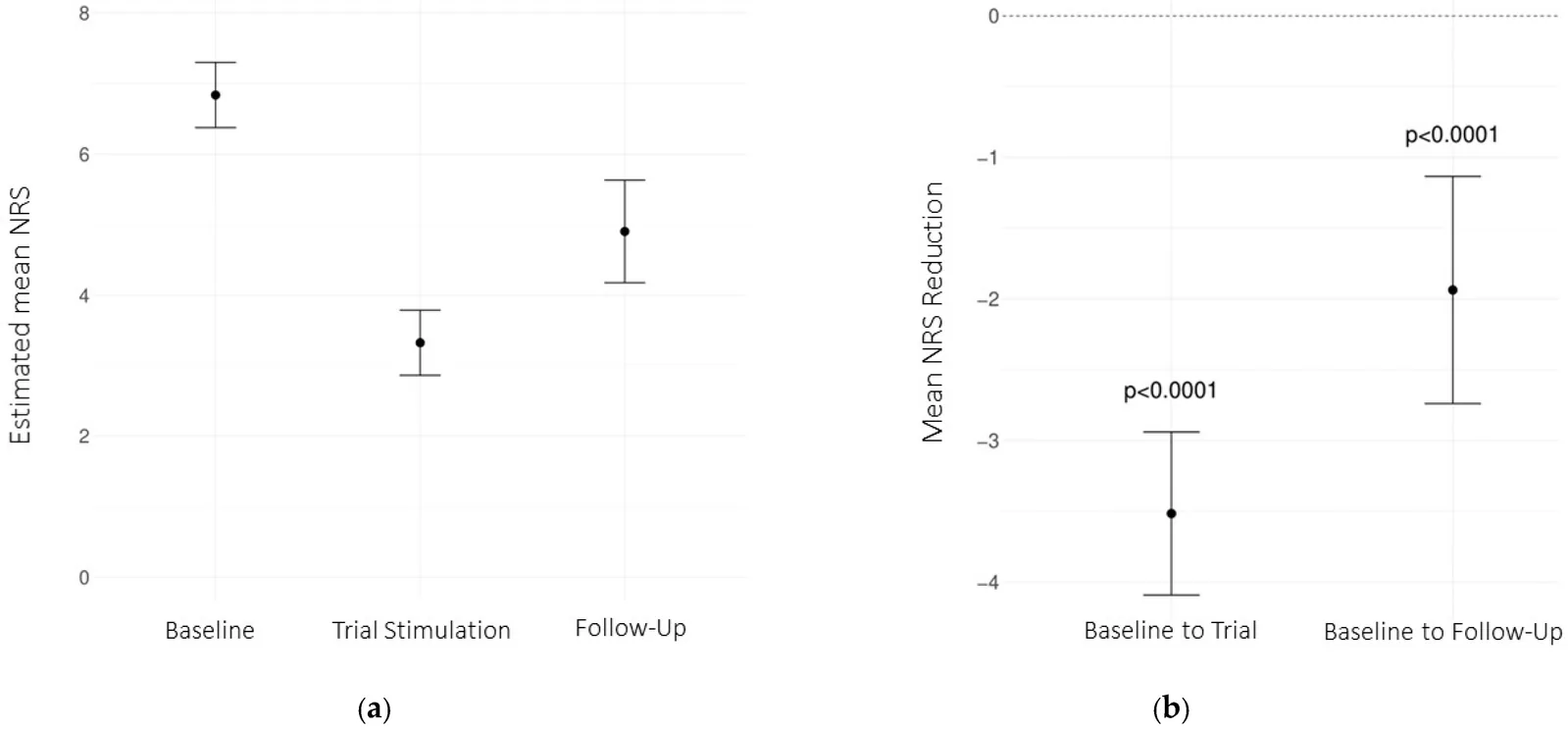
(**a**) Mean NRS Score at Baseline, during Trial Phase and at Follow-Up (**b**) NRS Reduction from Baseline to Trial Phase and Baseline to Follow-Up. *n* = 71, missing values at follow-up addressed by multiple imputation method. *y*-axis: estimated mean NRS = Numeric Rating Scale; range 0–10. *x*-axis: time points: Baseline; Trial Stimulation; Follow-Up; Whiskers: 95% confidence intervals; Dot: estimated marginal means. *y*-axis: mean NRS reduction, NRS = Numeric Rating Scale *x*-axis: Baseline to Trial: Difference between mean trial NRS and mean baseline NRS; Trial to Follow-Up: Difference between mean follow-up NRS and mean baseline NRS; Whiskers: 95% confidence intervals; Dot: estimated marginal means.

The mean NRS score at baseline, during trial phase and at follow-up for this group are depicted in Figure 2a. NRS reduction from baseline to trial phase was −3.52 [95% CI: −2.94, −4.09] (*p* < 0.0001) while NRS reduction from baseline to long-term follow-up was −1.94 [95% CI: −1.10, −2.74] (*p* < 0.0001). This equated to an NRS increase of 1.58 [95% CI: 0.780, 2.38] points during the mean follow-up period of 4.42 [95% CI: 3.53, 5.30] years (*p* < 0.0002).

NRS reduction between baseline and trial phase and baseline and long-term follow-up determined by multiple imputation method is given in Figure 2b.

In a sensitivity analysis excluding explanted patients before multiple imputation the mean NRS score at baseline, trial phase and follow-up was 6.87 [95% CI: 6.28, 7.45], 3.04 [95% CI: 2.45, 3.62] and 4.60 [95% CI: 3.83, 5.37] respectively.

Therefore, NRS reduction from baseline to trial phase was −3.83 [95% CI: −3.12, −4.54] (*p* < 0.0001) while NRS reduction from baseline to long-term follow-up was −2.27 [95% CI: −1.4, −3.13] (*p* < 0.0001). This equated to an NRS increase of 1.56 [95% CI: 0.70, 2.43] points (*p* = 0.0005).

A further sensitivity analysis stratifying patients by implantation period (2001–2011, *n* = 16; 2012–2021, *n* = 55) showed a comparable increase in NRS from trial stimulation to long-term follow-up in both periods ((2001–2011): 1.81 [95% CI: 0.24, 3.39] vs. (2012–2021): 1.51 [95% CI: 0.63, 2.40]; entire cohort 1.58 [95% CI: 0.78, 2.38]).

### 3.5. Association of NRS During Trial Stimulation and Occurrence of SCS-Device Explantation

#### 3.5.1. Logistic Regression Model

In an univariable logistic regression model a one-point higher NRS-Score during trial stimulation was associated with an odd ratio (OR) of 1.34 for explantation of the device (95% CI: 1.00, 1.80) (*p* = 0.0517). In the multivariable model adjusted for sex, age, indication group, stimulation pattern, number of revisions, and baseline NRS, higher trial NRS showed an association with higher odds of explantation (OR 1.38, 95% CI: 1.01–2.04; *p* = 0.0458), which should be interpreted cautiously given the limited number of explantation events. Therefore, after adjustment for the included clinical covariates, one-point increase in trial NRS was associated with approximately 38% higher odds of explantation. None of the remaining covariates were significantly associated with explantation in the full adjusted model. The corresponding odds ratios and *p*-values are given in Supplementary Table S4.

#### 3.5.2. Cox Regression Model

In an univariable Cox proportional hazards model higher trial NRS was associated with an increased hazard of explantation. (HR = 1.26, 95% CI: 1.00–1.59, *p* = 0.0460).

A multivariable model, adjusted for sex, age, indication group, stimulation pattern, number of revisions or re-operations, and baseline NRS, showed that higher trial NRS was associated with increased explantation risk, although this did not reach conventional statistical significance after adjustment (HR = 1.25, 95% CI: 1.00–1.60, *p* = 0.0541). None of the remaining covariates showed a statistically significant association with explantation in the multivariable model. The corresponding hazard ratios and *p*-values are given in Supplementary Table S5.

#### 3.5.3. Kaplan-Meier Curves

Kaplan-Meier curves were constructed based on two groups defined by the NRS score at trial stimulation. Patients were categorized into two groups: low NRS (0≤ and <4) (*n* = 41) and high NRS (4≤ and ≤8) (*n* = 30).

A total of 21 patients were explanted. In the low trial NRS group 8 patients were explanted after a median of 56.3 months (IQR: 34.3–74.9 months) whereas 13 patients in the high trial NRS group underwent explantation after a median of 38.6 months (IQR: 26.2–48.0 months).

Therefore, patients in the higher NRS group experienced a faster and more frequent progression to explantation compared to those with lower NRS scores (log-rank test, *p* = 0.02).

**Figure 3:**
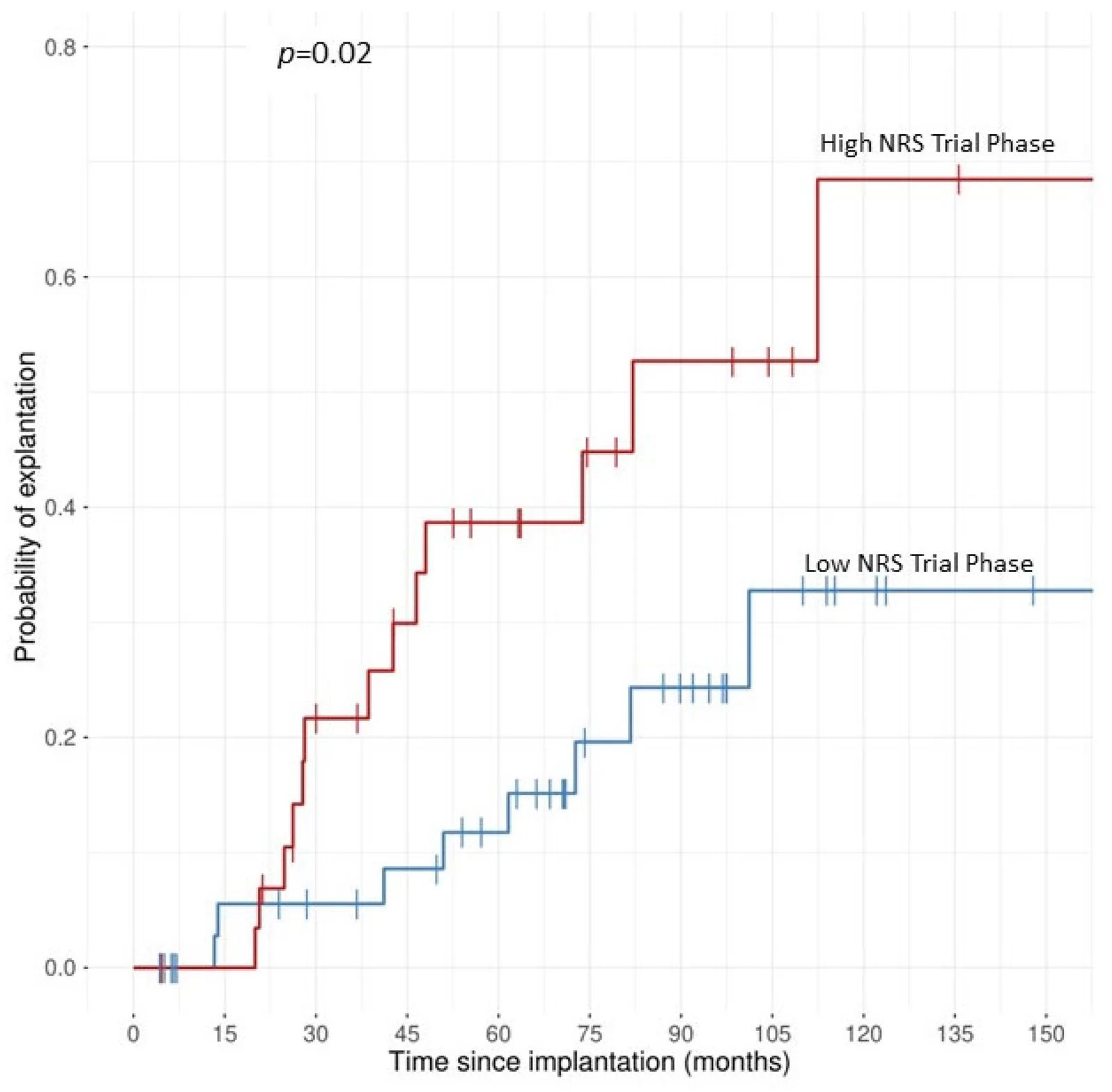
Kaplan-Meier curves for Probability of Explantation in a Low and High NRS Group during Trial Phase. Blue: low mean NRS during trial phase (0≤ and <4) (*n* = 41); Red: high mean NRS during trial phase (4≤ and ≤8) (*n* = 30).

Kaplan-Meier curves for the low and high NRS group are depicted in Figure 3.

#### 3.5.4. ROC-Curves

**Figure 4:**
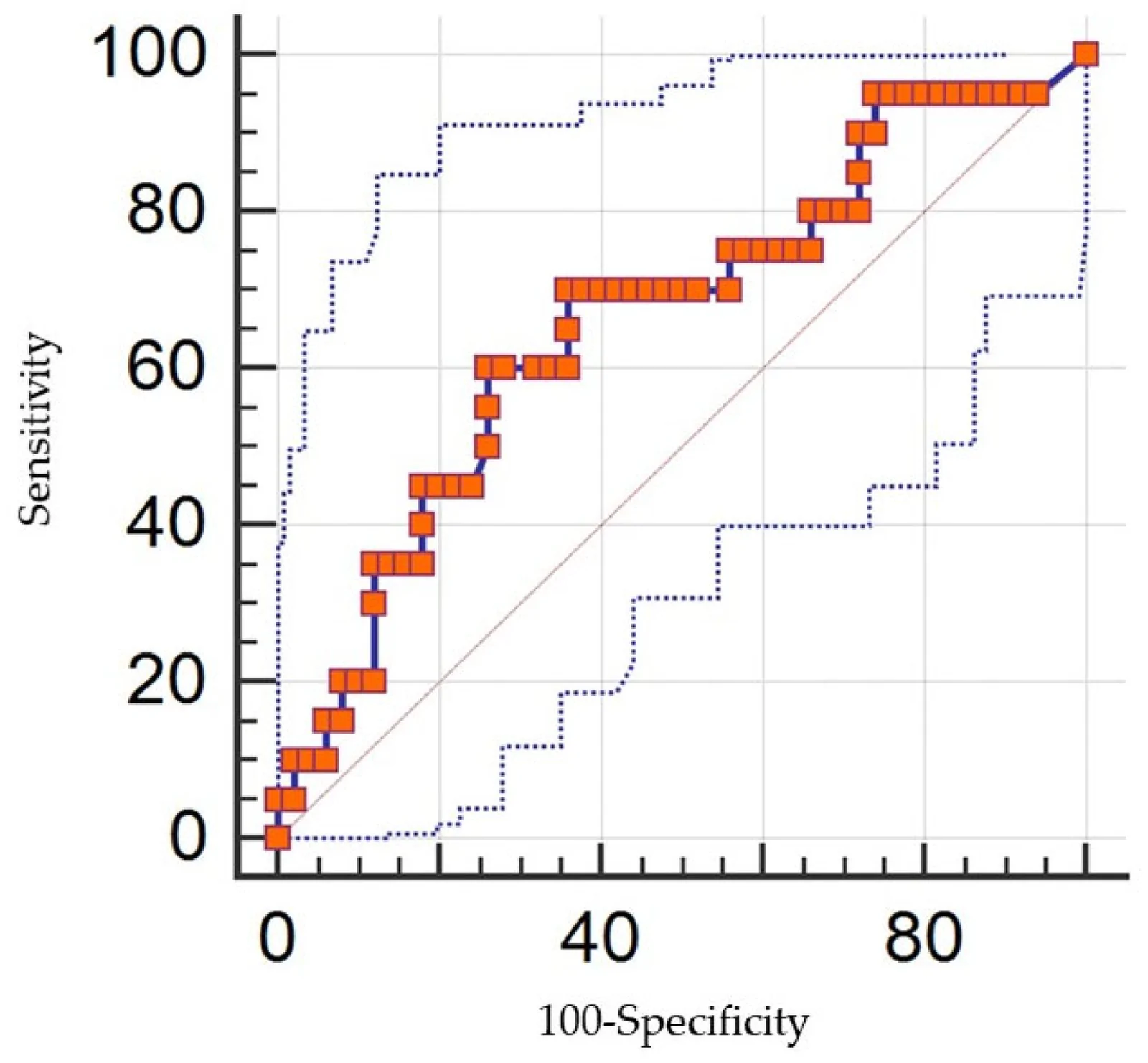
Receiver operating characteristic (ROC) curves of percentage NRS reduction from baseline to trial phase as Predictor for Explantation. The red line represents percentage reduction in NRS level from baseline to trial phase. Dotted lines are 95% confidence intervals. Area under the curve (AUC) = 0.66.

Cut-off values of percentage NRS reduction from baseline to trial phase for discriminating between patients with and without system explantation were determined by ROC-curve analysis. The corresponding ROC-curve is shown in Figure 4.

ROC analysis suggested modest discrimination between patients with and without subsequent explantation (AUC = 0.660; *p* = 0.0280). The cut-off value with the maximum accuracy was an NRS reduction of ≤39% from baseline to trial phase.

A summary of all values obtained for this cut-off including sensitivities and specificities is given in Table 2.

The negative predictive value (NPV) of a ≥39% reduction of NRS from baseline for explantation is 80%.

### 3.6. Oral Morphine Milligram Equivalents (OMME) at Baseline and Follow-Up

Mean OMME at baseline and follow-up in all included patients (*n* = 71) were 76.9 mg ± 74.3 and 80.7 mg ± 83.1 respectively. Mean OMME did not differ significantly between baseline and follow-up. (*p* = 0.706).

When excluding explanted patients (*n* = 50) Mean OMME at baseline and follow-up were 84 mg ± 72 and 70 mg ± 70 respectively. Mean OMME did not differ significantly between baseline and follow-up. (*p* = 0.550).

## 4. Discussion

The current study compares pain levels during SCS trial phase to long-term results.

We observed a significant loss of efficacy in pain reduction from SCS-trial stimulation to follow-up. However, the NRS reduction of 1.94 at follow-up was still statistically significant and corresponds to usual definitions of clinical efficacy [17].

Furthermore, mean OMME remained stable from baseline to follow-up despite the observed attrition in reported pain reduction.

The pain reduction at follow-up compared to baseline reported here is less than that reported by other authors [6,7,8,9,10]. Here NRS reductions from baseline between 2.3 and 5 points were reported at follow-up between 1 and 10.6 years.

Comparability of these studies is reduced due to varying follow-up periods and indications. Furthermore, the studies quoted above did not include pain levels in patients, in whom the SCS-system was explanted, which leads to an overestimation of efficacy.

There is a paucity of studies comparing initial pain reduction after SCS- implantation with pain levels at follow-up. A single retrospective study reported a significant increase in VAS-score of 1.95 from 1 month after implantation to 2 years after implantation [11]. This compares well to the results of the current study, where a significant increase in NRS score by 1.58 points was also observed from trial-stimulation to follow-up.

Loss of efficacy of SCS-therapy is also partially reflected in explantation rates as insufficient pain reduction constitutes the most frequent reason for explantation of the SCS device. The explantation rate of 29.6% at 4.1 ± 2.4 years in the current study population is similar to those reported by other authors in larger samples [12,13]. Because insufficient pain relief may contribute to the decision for device explantation, longitudinal pain outcomes and explantation are not entirely independent endpoints. Patients undergoing explantation may represent a subgroup with more pronounced treatment failure, and inclusion of their pre-explantation NRS measurements may therefore contribute to the magnitude of the observed loss of efficacy over time. However, in a sensitivity analysis excluding explanted patients before multiple imputation, the estimated long-term change in NRS was consistent and the increase in NRS score from trial to follow-up remained statistically significant. Thus, the observed long-term attrition of NRS-reduction was not solely attributable to explantation-related treatment failure.

While long-term loss of efficacy of SCS-therapy is increasingly well documented [4,5,11,18,19,20], the reasons for this loss remain unclear. A prospective, randomized, sham-control double blind cross-over trial of SCS in FBSS demonstrated, that 12 weeks of sham treatment can produce a 3-point VAS-reduction from baseline [21]. In a randomized controlled trial of SCS and physiotherapy versus physiotherapy alone in CRPS patients, the initially significant difference in pain reduction between the groups was lost by year three [3,4,5]. Based on these findings, an initial placebo effect, augmented by the invasive and costly nature of the intervention, may be relevant. This effect may however diminish with time.

Furthermore, other mechanisms such as increase of pain level over time through disease progression cannot be excluded. As the decision for IPG implantation is often based on reported pain reduction during the trial phase, overreporting of pain decrease during the trial phase may also contribute.

Other authors have evaluated the predictive potential of pain reduction during trial stimulation for long-term SCS-treatment success. Weinand et al. found a positive predictive value (PPV) of 86% for treatment success [22]. Williams et. al. reported a strong association between pain relief <50% during trial phase and a negative result (pain relief ≤50%) at ≥6 months [23]. Orhurhu et al. found that a pain score ≤4.9 during trial had a greater than 50% probability for successful implantation [24].

On the other hand, the utility of trial stimulation for prediction of treatment success is called into question by a randomized controlled trial that found no difference in NRS levels between a group of patients implanted after successful trial stimulation as compared to a group implanted after a screening without trial at a 6-month [25] and 36- month [16] follow-up. There was also no difference in number of device explants at 36 months.

While it is desirable to predict treatment success in SCS-treatment, it is equally important to predict treatment failure in terms of explantation. As loss of efficacy is the most prevalent reason for SCS-device removal, explantation can be considered a hard endpoint in terms of treatment outcome [12,13]. The studies quoted above had relatively short observation periods between 6–36 months, considering that risk of treatment failure reflected in cumulative explantation risk due to diminished pain relief only starts to rise at 3 years implantation time [13].

In the current study patients with high NRS during trial phase (NRS 4–8) showed earlier and more frequent explantation than those with lower trial NRS scores.

Also, higher trial NRS showed a borderline association with higher odds of explantation in the multivariable logistic regression.

The limitations of this study include its retrospective design, relatively small sample size, inhomogeneous underlying chronic pain conditions, and the limited number of explantation events relative to the number of covariates considered in the multivariable models; these adjusted analyses should therefore be considered exploratory and interpreted with caution.

Patients with available baseline and trial data differed from excluded patients in the distribution of indications, which may limit generalisability.

Missing NRS values at baseline, trial and during follow-up constitute a further limitation. Missing follow-up NRS values were addressed by multiple imputation under a Missing At Random (MAR) assumption, conditional on observed patient characteristics and the available NRS history. The 20-year observational period introduced substantial variation in documentation practices and data completeness over time, which provides a plausible administrative source of missingness. Nevertheless, the MAR assumption cannot be verified from the observed data. In particular, a Missing Not At Random (MNAR) mechanism cannot be excluded for follow-up diary non-response, as patients dissatisfied with treatment may have been less likely to respond, potentially biasing outcomes in a favorable direction; women were also underrepresented among diary returners. Missingness related to explantation may likewise be informative because explantation can be associated with insufficient pain relief or treatment failure. However, sensitivity analyses excluding explanted patients before multiple imputation yielded comparable long-term estimates.

The 20-year recruitment period should also be considered, as it introduces potential heterogeneity due to substantial changes in SCS hardware, stimulation paradigms and patient selection. Although sensitivity analyses stratified by implantation era showed comparable long-term pain trajectories and explantation-free survival, the small number of patients implanted before 2012 limits the power to detect era-specific differences, and residual confounding by temporal changes in practice cannot be excluded.

The absence of a control arm is an additional limitation. The reduction in pain intensity observed during the SCS trial phase may have been inflated by expectation effects in the absence of blinding. Without a comparator, the magnitude of any such effect cannot be estimated.

Parameters other than pain levels, important for quantification of treatment success in chronic pain conditions, such as scores evaluating physical and emotional functioning [26], were not available for analysis. This is an important limitation, as pain level alone does not fully capture therapeutic response to SCS [27].

Finally, as the analysed patients were predominantly treated with tonic stimulation, comparability to patient groups treated with non-paraesthesia-based stimulation modalities may be limited.

## 5. Conclusions

In the current study, pain reduction diminished between SCS trial stimulation and long-term follow-up. Higher pain levels during trial stimulation showed a possible association with subsequent device explantation.

In view of these results, it is necessary to inform patients eligible for SCS about the possible loss of efficacy over time and to limit the patient expectation that initial pain reduction during the trial phase can be maintained permanently. Transparent communication regarding the potential for waning efficacy may help align expectations and reduce dissatisfaction should pain relief diminish.

Trial-phase pain level is potentially associated with risk of device explantation. However, its value for estimation of explantation risk has to be investigated further.

Future large, prospective trials incorporating multidimensional outcome measures are needed to better define predictors of long-term success and failure in SCS therapy.

## Figures and Tables

**Table 1:** Baseline Patient Characteristics of Included Patients, Excluded Patients and entire Cohort.

	Total Cohort (*n* = 171)	Included Patients (*n* = 71)	Excluded Patients (*n* = 84)	Included Patients vs. Total Cohort	Included Patients vs. Excluded Patients
**Age at implantation, years** **(mean ± SD)**	55.4 ± 14.5	53.1 ± 13.0	55.4 ± 15.2	*p* > 0.05	*p* > 0.05
**Gender, female, *n* (%)**	83 (48.5)	35 (49.3)	42 (50)	*p* > 0.05	*p* > 0.05
**Mean baseline NRS-score (±SD)**	n.a.	6.8 ± 1.5	n.a.		
**Implant Indication, *n* (%)**					
**Failed Back Surgery Syndrome (FBSS)**	76 (44.4)	39 (54.9)	29 (34.5)	*p* > 0.05	*p* = 0.0270
**Complex Regional Pain Syndrome (CRPS)**	23 (13.5)	8 (11.3)	15 (17.9)	*p* > 0.05	*p* > 0.05
**Polyneuropathy (PNP)**	11 (6.4)	5 (7)	2 (2.4)	*p* > 0.05	*p* > 0.05
**Chronic low back pain with leg pain**	25 (14.6)	11 (15.5)	13 (15.5)	*p* > 0.05	*p* > 0.05
**Other**	36 (21.1)	8 (11.3)	25 (29.8)	*p* > 0.05	*p* = 0.0250

Numeric Rating Scale (NRS); Data is given as percentages or mean ± standard deviation as appropriate. Not available (n.a.)

**Table 2:** Synopsis of the predictive Numeric Rating Scale (NRS) percentage reduction during trial phase for System Explantation.

Area Under ROC Curve (AUC)	*p*-Value (Area = 0.5)	Cut-Off Value NRS-Reduction Trial Phase (%)	Sensitivity, (%)	Specificity, (%)	PPV, (%)	NPV, (%)
0.66	0.028	≤39	57	74	48	80

Synopsis of the predictive NRS percentage reduction during trial phase differentiating patients with system explantation from non-explantation patients, including sensitivities, specificities, positive predictive value (PPV) and negative predictive value (NPV). Cut-off value NRS-Reduction Trial Phase (%), Sensitivity (%), Specificity (%), PPV (%), NPV (%).

## Data Availability

De-identified patient data and data dictionary that underlie the results reported in this manuscript can be made available to investigators for research purposes on a case-by-case basis with publication and after approval of research proposal by all co-authors. A signed data access agreement will be required. Requests for access to data should be addressed to the corresponding author at sibylle.pramhas@meduniwien.ac.at.

## Supplementary Materials

The following supporting information can be downloaded at: https://www.mdpi.com/article/10.3390/jcm15218294/s1, Supplementary Material S1: STROBE Checklist, Supplementary Material S2: Additional Statistical Methods; Figure S1: Distribution of Trial-Stimulation NRS and Data-Driven Cut-Off Evaluation Using Maximally Selected Rank Statistics; Figure S2: Model-based NRS estimates across analytical approaches; Figure S3: Distribution of Observed and Imputed NRS Values by Patient Group and Time Point; Figure S4: Calibration of Imputed Versus Observed Long-Term NRS in the Mask-And-Recover Experiment; Figure S5: Kaplan-Meier curves for Probability of Explantation in Included and Excluded Patients; Table S1: Level of Lead Tip Placement; Table S2: Mean NRS at Baseline, Trial Stimulation and Long-Term Follow-Up in Indications FBSS and ‘Other’ compared to ‘non-FBSS’ and ‘non-Other’; Table S3: Baseline characteristics by Long-Term Follow-Up NRS Diary Return; Table S4: Multivariable Firth logistic regression model, Table S5: Multivariable Firth Cox proportional hazards model.

## Abbreviations

The following abbreviations are used in this manuscript:

SCS	Spinal Cord Stimulation
NRS	Numeric Rating Scale
ROC	Receiver Operating Characteristics
FBSS	Failed Back Surgery Syndrome
CRPS	Complex Regional Pain Syndrome
IPG	Implantable Pulse Generator
MI	Multiple Imputations
MAR	Missing-At-Random
MNAR	Missing-Not-At-Random
OMME	Oral Morphine Milligram Equivalent
