# Supplementary material for: Sustainability of Pain Reduction from Trial Stimulation to Long-Term Follow-Up in Chronic Pain Patients with Spinal Cord Stimulation: A Retrospective Analysis
Source: J Clin Med. 2026 Oct 24;15(21):8294. doi: 10.3390/jcm15218294 (PMC13763605; doi:10.3390/jcm15218294)
Supplement: Supplementary file 1 [file jcm-15-08294-s001.zip › jcm-4564216-supplementary.pdf]

Supplementary Material S1. STROBE Statement—Checklist of items that should be included in reports of Cohort Studies

	Item No	Recommendation
**Title and abstract**	1	(*a*) Indicate the study's design with a commonly used term in the title or the abstract **p.1**
		(*b*) Provide in the abstract an informative and balanced summary of what was done and what was found **p.1**
**Introduction**		
Background/rationale	2	Explain the scientific background and rationale for the investigation being reported<br>**p.2**
Objectives	3	State specific objectives, including any prespecified hypotheses **p.2**
**Methods**		
Study design	4	Present key elements of study design early in the paper **p.2, p.3**
Setting	5	Describe the setting, locations, and relevant dates, including periods of recruitment, exposure, follow-up, and data collection **p.3**
Participants	6	(*a*) Give the eligibility criteria, and the sources and methods of selection of participants. Describe methods of follow-up **p.4**
		(*b*) For matched studies, give matching criteria and number of exposed and unexposed **not applicable**
Variables	7	Clearly define all outcomes, exposures, predictors, potential confounders, and effect modifiers. Give diagnostic criteria, if applicable **p.4, p.5**
Data sources/ measurement	8*	For each variable of interest, give sources of data and details of methods of assessment (measurement). Describe comparability of assessment methods if there is more than one group **p.4**
Bias	9	Describe any efforts to address potential sources of bias **p.5, p.6, p.8 S4, S5, S6**
Study size	10	Explain how the study size was arrived at **p.5, p.6, Figure 1. (p.7)**
Quantitative variables	11	Explain how quantitative variables were handled in the analyses. If applicable, describe which groupings were chosen and why **p.4, p.5**
Statistical methods	12	(*a*) Describe all statistical methods, including those used to control for confounding **p.4, p.5**
		(*b*) Describe any methods used to examine subgroups and interactions **p.5**

		(*c*) Explain how missing data were addressed **p.4**
		(*d*) If applicable, explain how loss to follow-up was addressed **p.4**
		(*e*) Describe any sensitivity analyses **p.5**
**Results**		
Participants	13*	(a) Report numbers of individuals at each stage of study—eg numbers potentially eligible, examined for eligibility, confirmed eligible, included in the study, completing follow-up, and analysed **p.5, p.6**
		(b) Give reasons for non-participation at each stage **p.5, p.6**
		(c) Consider use of a flow diagram **p.7, Figure 1.**
Descriptive data	14*	(a) Give characteristics of study participants (eg demographic, clinical, social) and information on exposures and potential confounders **Table 1, p.6**
		(b) Indicate number of participants with missing data for each variable of interest **p.5, p.6, Figure 1**
		(c) Summarise follow-up time (eg, average and total amount) **p.6**
Outcome data	15*	Report numbers of outcome events or summary measures over time **p.8**
Main results	16	(*a*) Give unadjusted estimates and, if applicable, confounder-adjusted estimates and their precision (eg, 95% confidence interval). Make clear which confounders were adjusted for and why they were included **p.8, p.9, p.10, p.11, p.12, Figure 2, Figure 3, Figure 4**
		(*b*) Report category boundaries when continuous variables were categorized
		(*c*) If relevant, consider translating estimates of relative risk into absolute risk for a meaningful time period
Other analyses	17	Report other analyses done—eg analyses of subgroups and interactions, and sensitivity analyses **p.8, p.9, p.10, S4, S5, S6**
**Discussion**		
Key results	18	Summarise key results with reference to study objectives **p.13, p.14**
Limitations	19	Discuss limitations of the study, taking into account sources of potential bias or imprecision. Discuss both direction and magnitude of any potential bias **p.15**

Interpretation	20	Give a cautious overall interpretation of results considering objectives, limitations, multiplicity of analyses, results from similar studies, and other relevant evidence **p.14, p.15, p.16**
Generalisability	21	Discuss the generalisability (external validity) of the study result **p.15**
**Other information**		
Funding	22	Give the source of funding and the role of the funders for the present study and, if applicable, for the original study on which the present article is based **p.16**

Supplementary Material S2: Additional Statistical Methods

Supplementary Material S2.1 Justification of the Trial Stimulation NRS Cut-off for Time-to-Explantation Analyses (Figure S1.)

The cut-off was evaluated using maximally selected rank statistics. This approach examines all possible cut-points and identifies the threshold that maximizes the standardized log-rank statistic, corresponding to the strongest separation between time-to-event curves. The optimal cut-off was identified at a trial-stimulation NRS value of 4.1. Patients were therefore grouped into a low trial-stimulation NRS group (0-4) and a high trial-stimulation NRS group (>4) for the Kaplan-Meier analysis.

This cut-off-based analysis should be interpreted as exploratory, as the threshold was evaluated post-hoc using the available data.

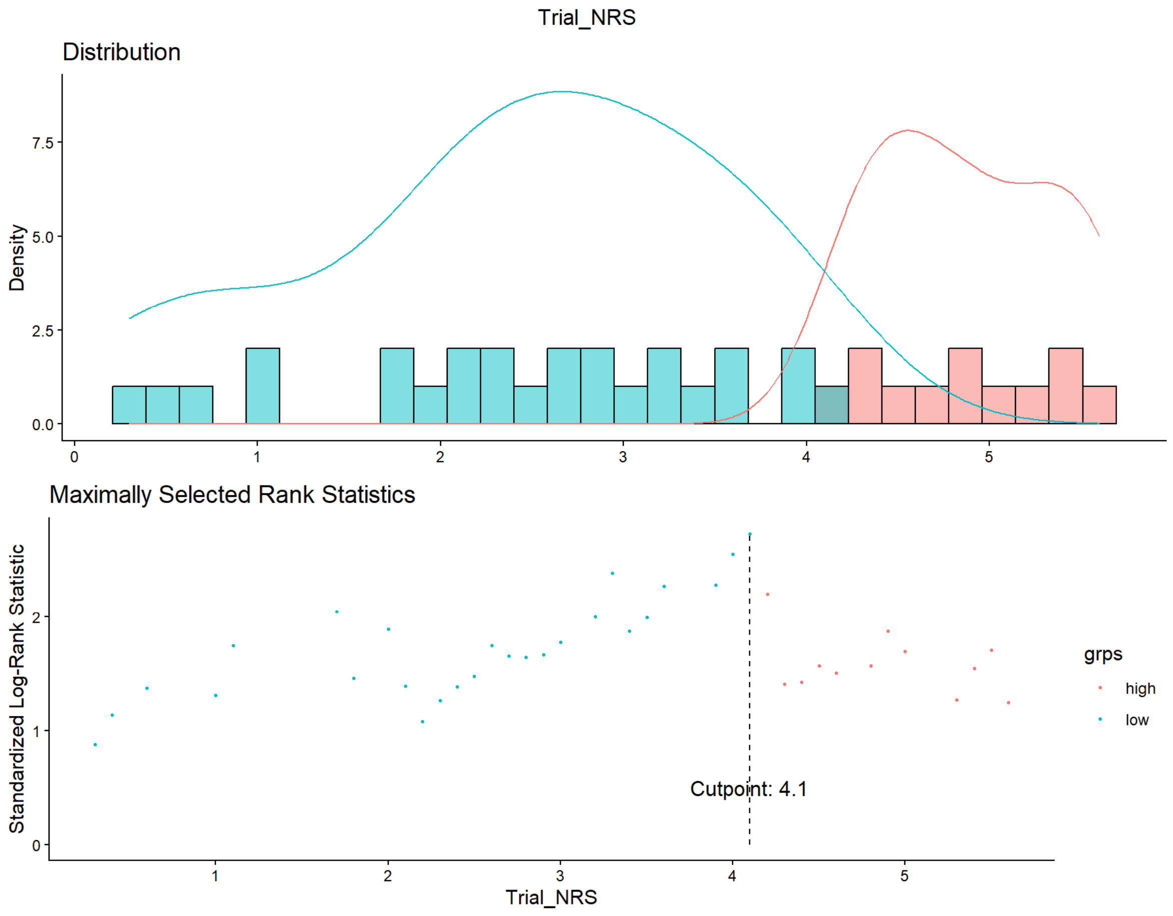

Figure S1. Distribution of Trial-Stimulation NRS and Data-Driven Cut-Off Evaluation Using Maximally Selected Rank Statistics.

Supplementary Material S2.2 Plausibility of the Missing at Random (MAR) Assumption for Missing Follow-up NRS Values (Figure 24)

The MAR assumption cannot be formally proven from the observed data, because it concerns the relationship between missingness and unobserved NRS values. However, MAR was considered a reasonable working assumption for the MI analysis because the imputation model conditioned on observed information that was related to both missingness and NRS outcomes, including baseline covariates and the available NRS history with lagged NRS values. Therefore, missing follow-up values were imputed conditional on observed patient characteristics and previous pain measurements, reducing the risk that missingness depended on unmodelled observed differences between patients.

Nevertheless, missingness after explantation was specifically considered as a potential source of MNAR missingness, since explantation may be related to insufficient pain relief, complications or treatment failure, and may therefore depend on unobserved or future NRS values. To assess the sensitivity of the findings to this potentially informative missingness mechanism, an additional analysis excluded explanted patients before imputation.

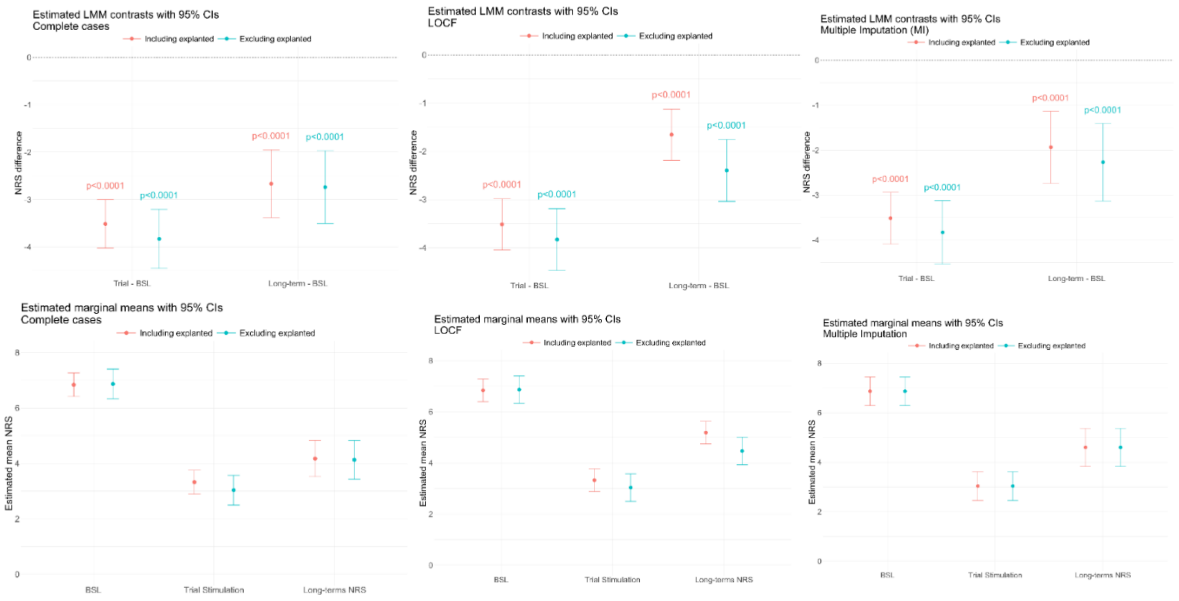

Figure S2. Model-based NRS estimates across analytical approaches. The upper row shows estimated pairwise contrasts in NRS between time points from linear mixed-effects models(LMM) based on complete cases, multiple imputation (MI) and last observation carried forward (LOCF). The lower row shows estimated marginal mean NRS values at each time point for the same three analytical approaches. Error bars indicate 95% confidence intervals.

Excluding explanted patients mainly affected the LOCF analysis, whereas complete-case and MI analyses yielded closely comparable long-term NRS estimates with and without explanted patients. This consistency suggests that the main conclusions were robust to the handling of potentially informative post-explantation missingness. For non-explantation-related missingness, additional exploratory checks did not indicate clear systematic differences in observed NRS trajectories between long-term responders and non-responders among non-explanted patients. This supported the plausibility of MAR for ordinary long-term non-response, conditional on baseline covariates and available NRS history.

Convergence and plausibility of the multiple imputation procedure were assessed using trace plots and density plots comparing observed and imputed values (Figure C). Density plots were generated by time point and by patient group; imputed values from all imputed datasets were pooled and compared with the observed distributions. Particular attention was given to the alignment between observed and imputed long-term follow-up NRS values, as this represented the primary follow-up endpoint. The imputed distributions showed good agreement with the observed data, supporting the plausibility of the imputation model under the assumed MAR framework.

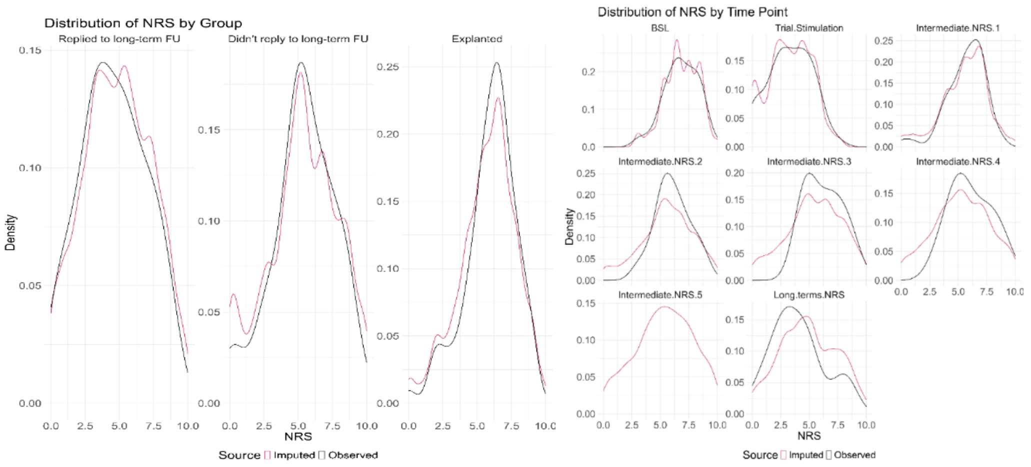

Figure S3. Distribution of Observed and Imputed NRS Values by Patient Group and Time Point.

In addition to visual diagnostics, a mask-and-recover experiment (Figure D) was conducted to evaluate imputation performance under an imposed MAR missingness mechanism, focusing specifically on long-term NRS as the primary follow-up endpoint. In this experiment, 90% of the observed long-term NRS values were artificially set to missing and then re-imputed using the same two-level normal imputation model. The original observed values were retained as reference values for comparison with the corresponding imputed values. Imputation performance was quantified using bias, defined as the mean difference between imputed and observed values, and root mean squared error (RMSE), defined as the average magnitude of imputation error. This analysis was intended as a diagnostic stress test of the imputation model. The mask-and-recover analysis showed very small average prediction errors, with a bias of 0.00039 and an RMSE of 0.00196. Under the imposed missingness mechanism, this indicated that the imputation model recovered artificially masked long-term NRS values with negligible average bias and high numerical agreement.

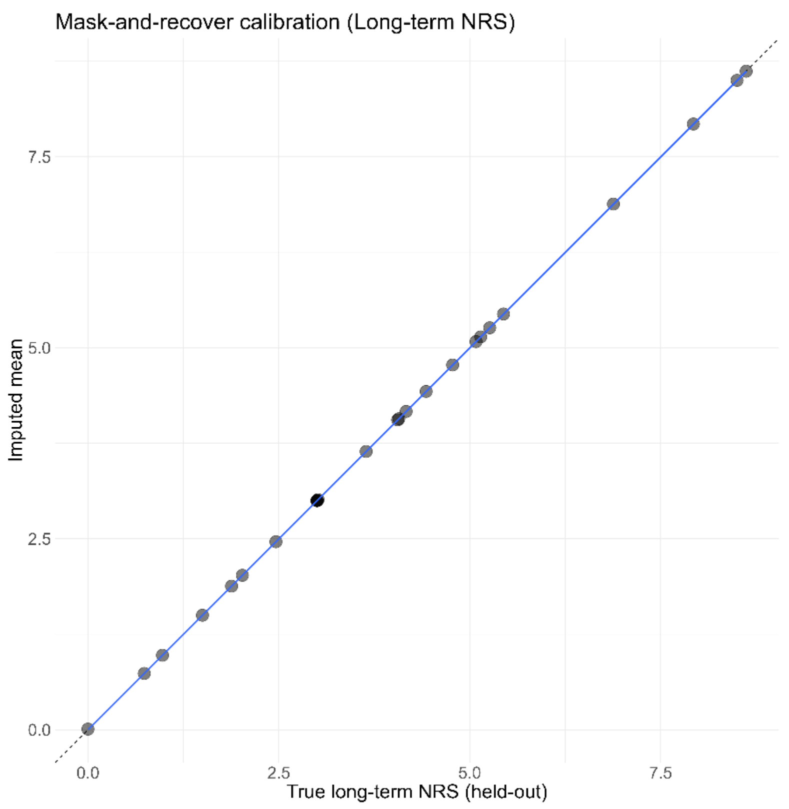

Figure S4. Calibration of Imputed Versus Observed Long-Term NRS in the Mask-And-Recover Experiment.

Together with the density and trace diagnostics, these results supported the stability and plausibility of the imputation procedure for the primary follow-up endpoint under the MAR framework. Nevertheless, MAR cannot be verified from the observed data alone. Follow-up diary non-response may still include an MNAR component if patients with poorer treatment satisfaction were less likely to respond, potentially resulting in more favourable long-term outcome estimates. Therefore, MI results were interpreted in relation to complete-case, LOCF and sensitivity analyses excluding explanted patients. The close agreement of the main estimates across these approaches supported the robustness of the findings, while acknowledging that MNAR mechanism

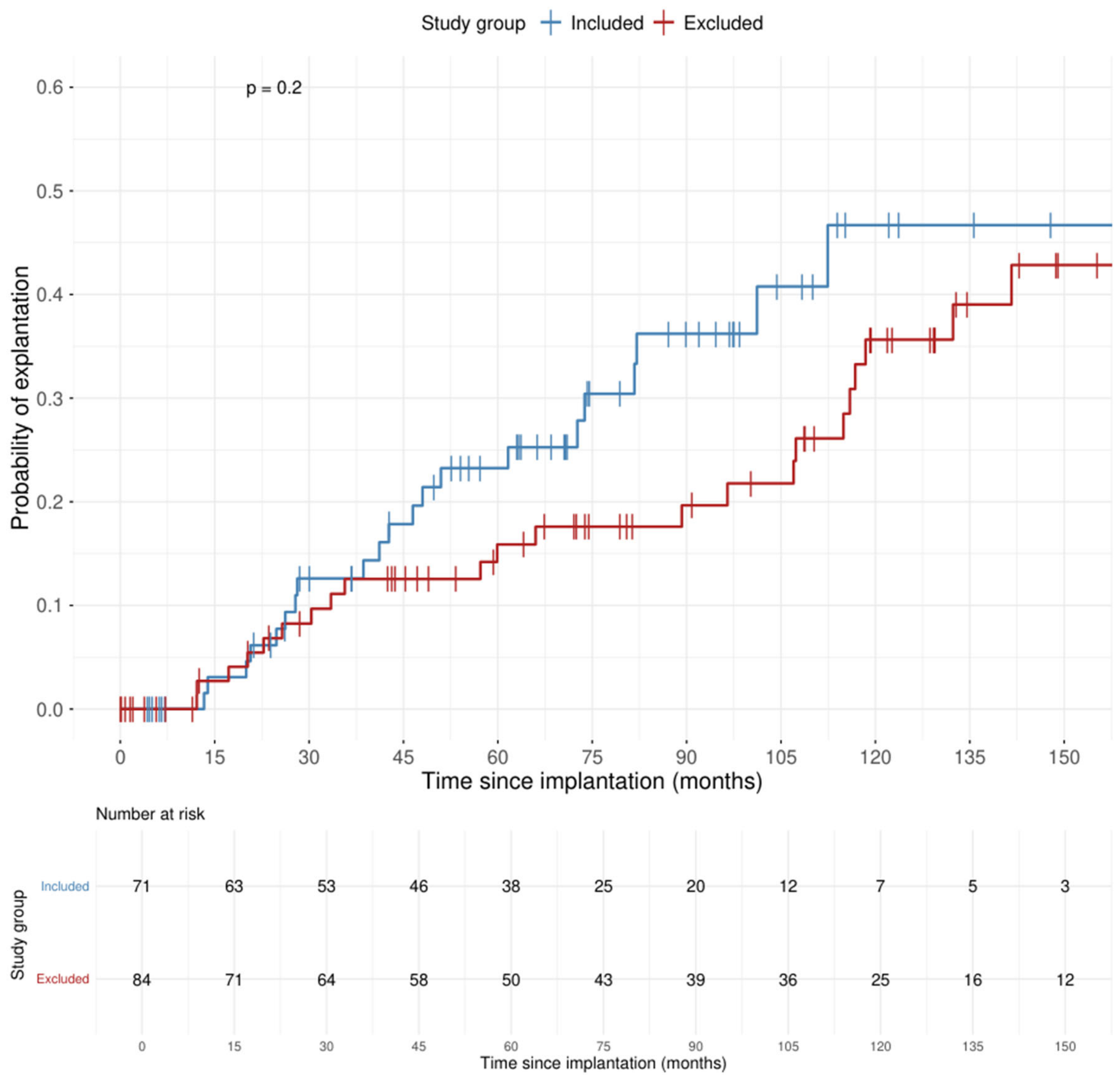

Supplementary Figure S5: Kaplan-Meier curves for Probability of Explantation in Included and Excluded Patients. Blue line: Included patients (n=71), Red line: Excluded patients (n=84), x-axis: Time since implantation of IPG (months), y-axis: Probability of explantation, Patients without explantation were censored at their last available follow-up

Supplementary Table S1: Level of Lead Tip Placement

Level of Lead Tip Placement	n
C5	2
C6	1
C7	1
Th2	1
Th6	1
Th7	10
Th8	29
Th9	17
Th10	7
Th12	1
L1	1

Lead placement is reported in included patients (n=71)

Supplementary Table S2. Mean NRS at Baseline, Trial Stimulation and Long-Term Follow-Up in Indications FBSS and 'Other' compared to 'non-FBSS' and 'non-Other'

Variables	Overall (n = 71)	FBSS (n = 39)	Other (n = 8)	p (FBSS vs. non-FBSS)	p (Other vs. non-Other)
**Baseline NRS**				**0.21**	**0.77**
Mean	6.84	6.69	6.6		
SD	1.5	1.44	1.93		
**Trial stimulation NRS**				**0.24**	**0.05**
Mean	3.32	3.59	2.04		
SD	1.89	1.8	1.87		
**Long-term FU NRS**				**0.59**	**0.31**
Mean	4.02	4.12	3.36		
SD	2.33	1.69	3.23		
**BSL - Trial**				**0.10**	**0.15**
Mean	3.52	3.1	4.56		
SD	2.13	2.04	2.47		
**BSL - Long-term FU**				**0.91**	**0.34**
Mean	2.5	2.37	3.21		
SD	2.25	1.9	2.52		

Supplementary Table S3. Baseline characteristics by Long-Term Follow-Up NRS Diary Return

Variables	Overall (n = 71)	Diary not returned (n = 43)	Diary returned (n = 28)	p-value
**Sex**				**0.0366**
**Female**	**35 (49.3 %)**	**26 (60.47 %)**	**9 (32.14 %)**	
**Male**	**36 (50.7 %)**	**17 (39.53 %)**	**19 (67.86 %)**	
**Indication**				**0.0606**
**Failed back surgery**	**39 (54.93 %)**	**27 (62.79 %)**	**12 (42.86 %)**	
**CRPS**	**8 (11.27 %)**	**5 (11.63 %)**	**3 (10.71 %)**	
**PNP**	**5 (7.04 %)**	**3 (6.98 %)**	**2 (7.14 %)**	
**Chronic low back pain with leg pain**	**11 (15.49 %)**	**7 (16.28 %)**	**4 (14.29 %)**	
**Other**	**8 (11.27 %)**	**1 (2.33 %)**	**7 (25 %)**	
**Age**				**0.0845**
**Mean**	**53.06**	**55.3**	**49.61**	
**SD**	**13**	**13.47**	**11.64**	
**Baseline NRS**				**0.2415**
**Mean**	**6.84**	**7.04**	**6.53**	
**SD**	**1.5**	**1.32**	**1.72**	

Supplementary Table S4. Multivariable Firth Logistic Regression Model

Variable	OR: 95% CI	p-value
NRS at trial stimulation	1.38: [1.01, 2.04]	0.0458
Sex: male	ref	
Sex: female	2.41: [0.78, 8.07]	0.1277
Age, per year	1.00: [0.95, 1.05]	0.9550
Indication: FBSS/CLBP with leg pain	ref	
Indication: CRPS/PNP/Other	0.32: [0.07, 1.15]	0.0814
Number of revisions/re-ops	0.71: [0.27, 1.32]	0.4701
Baseline NRS	0.87: [0.58, 1.32]	0.5146
Stimulation: tonic	ref	
Stimulation: high-frequency	0.56: [0.12, 2.25]	0.4207

OR = odds ratio; CI = confidence interval. Model fitted using Firth penalized logistic regression. Confidence intervals and p-values are based on profile likelihood.

Supplementary Table S5. Multivariable Firth Cox proportional hazards model

Variable	HR: 95% CI	p-value
NRS at trial stimulation	1.25: [1.00, 1.60]	0.0541
Sex: male	ref	
Sex: female	2.13: [0.86, 5.58]	0.1037
Age, per year	1.03: [0.99, 1.07]	0.1780
Indication: FBSS/CLBP with leg pain	ref	
Indication: CRPS/PNP/Other	0.37: [0.09, 1.15]	0.0874
Number of revisions/re-ops	0.75: [0.34, 1.24]	0.3892
Baseline NRS	0.95: [0.67, 1.32]	0.7458
Stimulation: tonic	ref	
Stimulation: high-frequency	0.47: [0.11, 1.48]	0.2077

HR = hazard ratio; CI = confidence interval. Model fitted using Firth penalized Cox regression. Confidence intervals and p-values are based on profile likelihood.
